# Preparation of High Stability Pd/Ceramic/Ti-Al Alloy Composite Membranes by Electroless Plating

**DOI:** 10.3389/fchem.2020.00202

**Published:** 2020-04-07

**Authors:** Dongqiang Zhang, Jing Zhao, Ping Yang, Yanan Chen, Yiqun Fan

**Affiliations:** ^1^College of Petrochemical Technology, Lanzhou University of Technology, Lanzhou, China; ^2^State Key Laboratory of Materials-Oriented Chemical Engineering, College of Chemical Engineering, Nanjing Tech University, Nanjing, China

**Keywords:** *in situ* oxidation, Ti-Al alloy, ceramic, Pd composite membrane, hydrogen separation

## Abstract

High stability Pd/ceramic/Ti-Al alloy composite membranes were prepared by electroless plating. Ceramic membranes fabricated by an *in situ* oxidation method were used as an inter-diffusion barrier between the Pd layer and the Ti-Al alloy support of the membranes to prevent intermetallic diffusion. The stabilities of the ceramic membranes at high temperatures in an H_2_ atmosphere were investigated. The permeation performances and stabilities of the Pd/ceramic/Ti-Al alloy composite membranes were also studied. The results showed that the thickness, pore size, and microstructure of the ceramic membranes did not change significantly after the treatment in an H_2_ atmosphere at high temperatures, indicating that the ceramic membranes prepared by the *in situ* oxidation method were stable in an H_2_ atmosphere at high temperatures. The thickness of the Pd layer was ~13 μm. The hydrogen permeability and H_2_/N_2_ selectivity of the Pd composite membranes at 773 K were 2.13 × 10^−3^ mol m^−2^ s^−1^ Pa^−0.5^ and 600, respectively. In addition, the H_2_ flux, N_2_ flux, and H_2_/N_2_ selectivity of the composite membranes remained nearly constant over three heat cycles (under the same conditions), indicating that the structures of the Pd/ceramic/Ti-Al alloy composite membranes were stable.

## Introduction

Recently, hydrogen has gained immense attention as a clean energy carrier for various industrial applications, especially for the development of fuel cells (Lemus and Duart, 2010; Vesborg et al., 2015; Sengodan et al., 2018). Pd membranes are widely used for hydrogen production and separation because of their high permeance and excellent selectivity for hydrogen (Keuler et al., 2002). The thickness of the Pd membrane is inversely proportional to the permeability. Hence, Pd-based membranes are usually prepared in the composite form, consisting of a thin, dense Pd top layer on a porous support (Zhang et al., 2006). Ti-Al alloys are considered promising porous supports for Pd membranes because of their higher specific strength, lower density, good oxidation resistance at higher temperatures, and similar thermal expansion co-efficient to that of Pd (Ma et al., 2007). However, although Ti-Al alloy averts the diffusion at temperatures lower than 873 K, the metal element diffusion phenomena occur between Pd and Ti-Al alloys after heat treatment at 973 K for 40 h in the presence of pure hydrogen (Zhang et al., 2011).

An effective approach to prevent intermetallic diffusion is to develop an inter-diffusion barrier layer between the Pd membrane and the metal support. However, the inter-diffusion barrier layer must be stable under high-temperature oxidation and reduction atmospheres. Huang and Dittmeyer (2006) used TiO_2_, YSZ, and ZrO_2_ as a diffusion barrier between the Pd layer and the sintered-metal support of composite membranes. They indicated that YSZ was the most promising inter-diffusion barrier and showed better intermetallic diffusion prevention than ZrO_2_ and TiO_2_. Zhang et al. (2009) used two different methods (*in situ* oxidized metal and sol-gel-derived YSZ) to prepare intermetallic diffusion barriers between Pd layer and PSS support of Pd composite membranes. They found that both the inter-diffusion barrier layers showed effective intermetallic diffusion prevention and the membranes were stable at temperatures in the range of 773–873 K. However, only the YSZ inter-diffusion barrier layer could prevent diffusion at temperatures higher than 873 K. Mardilovich et al. (1998) carried out an *in situ* oxidation reaction prior to plating Pd/PSS membranes to form an oxide membrane layer on porous stainless steel to act as a diffusion barrier between Pd and PSS. They found that the membranes were stable to heat treatment at 623–723 K for 6,000 h. Shu et al. (1993) employed sputtering to prepare an ultrathin intermediate layer of titanium nitride between a Pd/Ag alloy membrane and a PSS substrate. The results showed that the membranes were stable at temperatures higher than 973 K. Zhang et al. (2012) fabricated TiO_2_ membranes as a diffusion barrier on a Ti-Al alloy support to avoid intermetallic diffusion and to smooth the Ti-Al alloy support. The electron probe microanalysis (EPMA) and energy-dispersive X-ray spectroscopy (EDX) cross-sectional line scans showed that the TiO_2_ layer was an effective inter-diffusion barrier and was stable after 40 h of heat treatment at 973 K in the presence of pure H_2_. However, the adhesion between the TiO_2_ layer and Ti-Al alloy supports at high temperatures should be improved.

In this study, the high-temperature stability of the ceramic/Ti-Al alloy support was investigated by the gravimetric method, gas flux measurements, EDX, X-ray diffraction, and scanning electron microscopy (SEM) in a hydrogen atmosphere. Then, Pd membranes were prepared on the surface of the ceramic/Ti-Al alloy support, and their morphologies, permeabilities, selectivities, and stabilities were investigated in detail.

## Experimental

### Preparation of Pd/Ceramic/Ti-Al Alloy Composite Membranes

Ti-Al alloy discs with a diameter of 34 mm, a thickness of 2.5 mm, and an average pore size of 6 μm were used as the support for preparing the thin Pd composite membranes. The porous Ti-Al alloy discs were prepared using the method reported by He et al. (2007).

Prior to preparing the membranes, the supports were cleaned with acetone in an ultrasonic bath for 2 h to remove surface dirt and grease and were then dried overnight at 423 K. The ceramic membranes were prepared on Ti-Al alloy discs by sintering the Ti-Al alloy discs under a static air atmosphere at 1,023 K for 2 h in an electric furnace at the heating and cooling rate of 2 K/min. The procedure for the preparation of ceramic membranes has been described in detail in Zhang et al. (2015).

Electroless plating was used to deposit the Pd membranes on porous ceramic/Ti-Al alloy supports (Alique et al., 2016). Prior to the preparation of the palladium membranes, the surface of the Ti-Al alloy supports was activated and sensitized to seed Pd nuclei (Keuler et al., 2002). The deposition of Pd membranes was carried out at 313 K, and the plating bath was changed after every 60 min until dense Pd membranes with an ideal thickness were obtained. Deionized water was applied to clean the surface of the palladium membranes immediately after electroless plating to remove the impurities adsorbed on the surface and pores of the Pd layer. The composite membranes were then dried overnight at 423 K for 12 h (Zhang et al., 2012).

### Characterization of the Composite Membranes

An electronic balance (Sartorius BS2202S, Germany) was used to monitor the weight of the ceramic membranes. XRD (Bruker D8 Advance diffractometer, with Cu- radiation) was used to examine the phase composition of the ceramic/Ti-Al alloy composite membranes. EDX (JSM, 5600, LV, Japan) was used to analyze the composition of the ceramic membrane surface. The surface and cross-section of the palladium and ceramic membranes were investigated by SEM (Quanta 200, Philip, USA). The gas permeation of the composite membranes at high temperatures was measured using a permeability apparatus. The ceramic/Ti-Al alloy composite membranes were placed in a stainless steel permeator. The permeate side was maintained at atmospheric pressure, while the retentate side was fed with H_2_ or N_2_. A back-pressure regulator was used to control the upstream pressure. A mass flow controller (Models D08-4D/ZM, Beijing Sevenstar Electronics Co., Ltd., Beijing, China) was used to measure the single gas flux once the temperature and pressure became constant.

## Result and Discussion

### Stability of Ceramic/Ti-Al Composite Membranes

The ceramic membranes were prepared on Ti-Al alloy supports by an *in situ* oxidation method at 1,023 K for 2 h in an air atmosphere followed by their heat treatment in an H_2_ atmosphere for 20 h at 773, 873, and 973 K, respectively. The stability test results of the membranes are summarized in Table 1. From the table, it is clear that the weight of the ceramic/Ti-Al alloys did not change when the heat treatment was carried out at temperatures lower than 973 K. This indicates that the oxide layers were stable at temperatures lower than 973 K. As can be seen from Table 2, the change in the N_2_ flux of the ceramic/Ti-Al alloy composite membranes after the heat treatment was negligible. This indicates that the heat treatment did not destroy the pore structure of the ceramic membranes and that the ceramic layers were stable at high temperatures.

**Table 1:** Percent of weight gain of ceramic/Ti-Al alloy composite membrane after treatment in H_2_ atmosphere at different temperatures for 20 h.

**Sample**	**1#**	**2#**	**3#**
m_fresh_/g	5.45	5.72	6.19
Oxidation condition	750°C/2 h	750°C/2 h	750°C/2 h
m_oxidation_/g	6.33	6.57	7.24
Reduction condition	500°C/H_2_/20 h	600°C/H_2_/20 h	700°C/H_2_/20 h
m_reduction_/g	6.33	6.57	7.24
Δm(m_reduction_-m_oxidation)_/g	0	0	0

**Table 2:** Percent of N_2_ flux change of ceramic/Ti-Al alloy composite membrane after treatment in H2 atmosphere at different temperatures for 20 h.

**Sample**	**1#**	**2#**	**3#**
ΔP/MPa	0.03	0.03	0.03
J_fresh_/m^3^·m^−2^·h^−1^	239.58	258.66	260.78
Oxidation condition	750°C/2 h	750°C/2 h	750°C/2 h
J_oxidation_/m^3^·m^−2^·h^−1^	24.59	33.50	23.75
Reduction condition	500°C/H_2_/20 h	600°C/H_2_/20 h	700°C/H_2_/20 h
J_reduction_/m^3^·m^−2^·h^−1^	20.35	36.04	20.35
ΔJ(J_reduction_- J_oxidation_)/m^3^·m^−2^·h^−1^	−4.24	2.54	−3.40

**Figure 1:**
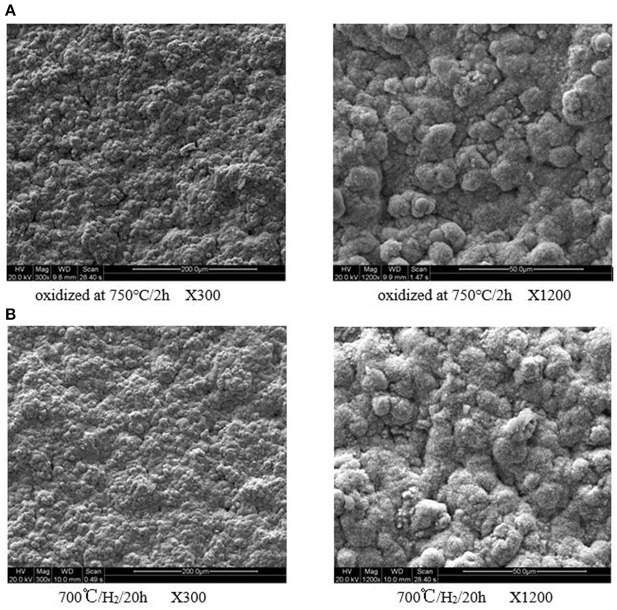
**(A)** Oxidized at 750°/2 h X300. **(B)** 700°/H_2_/20 h X300. **(C)** Oxidized at 750°/2 h X1200. **(D)** 700°/H_2_/20 h X1200. Surface SEM micrographs of the ceramic/Ti-Al alloy composite membrane in H2 atmosphere at different temperature for 20 h.

The surface SEM images of the ceramic/Ti-Al alloys are shown in Figure 1. The oxidation layer was evident, and no surface damages were observed even after the heat treatment at 973 K for 20 h in an H_2_ atmosphere. Hence, the ceramic layer prepared by *in situ* oxidation was stable in an H_2_ atmosphere. This is consistent with the weight and N_2_ flux measurement results.

**Figure 2:**
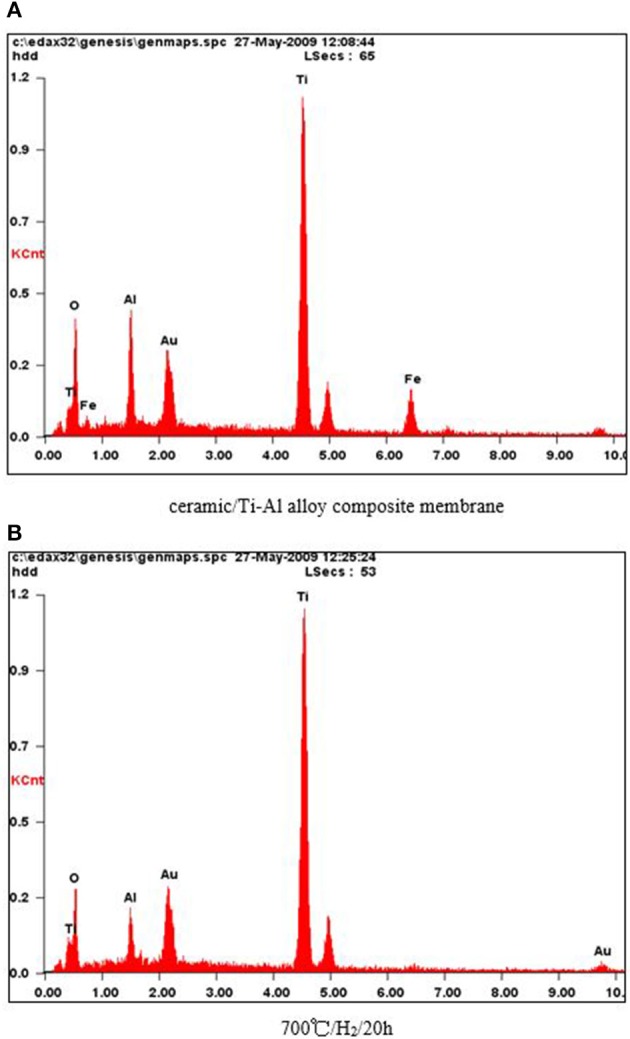
**(A)** Ceramic/Ti-Al alloy composite membrane. **(B)** 700°/H_2_/20 h. EDX characterization of the ceramic/T i-Al alloy composite membrane after treatment in H_2_ atmosphere at different temperature for 20 h.

In order to investigate the effect of heat treatment on the elemental composition of the surface of the Ti-Al alloy support, an EDX analysis of the membranes was carried out. As shown in Figure 2, oxygen was present on the support surface even after the heat treatment at 973 K in an H_2_ atmosphere for 20 h, indicating that the ceramic layer prepared by *in situ* oxidation was stable at higher temperatures in an H_2_ atmosphere.

**Figure 3:**
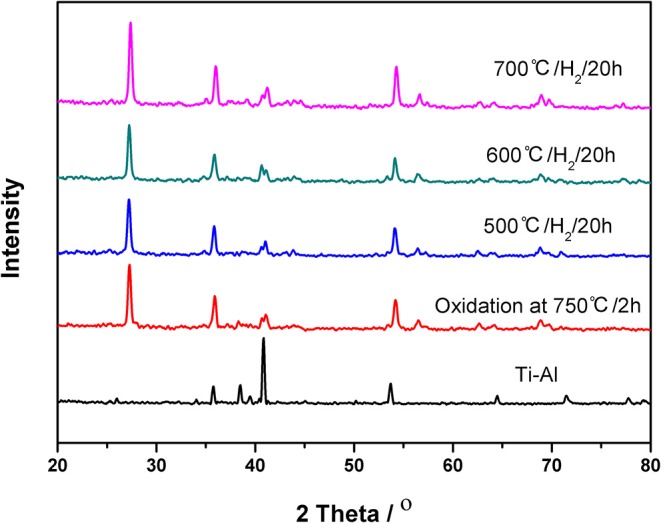
XRD patterns of the ceramic/Ti-Al alloy composite membrane in H_2_ atmosphere at different temperature for 20 h.

The XRD patterns of the ceramic/Ti-Al alloy membrane are shown in Figure 3. The characteristic peak keeps constant even after the heat treatment in an H_2_ atmosphere for 20 h at 773, 873, and 973 K, indicating that the ceramic/Ti-Al alloy composite membranes were highly stable to high temperatures in an H_2_ atmosphere. These results are consistent with the EDX results shown in Figure 2.

The results of gravimetric, gas flux, SEM, EDX, and XRD analyses show that the ceramic layer prepared by the *in situ* oxidation method is a promising inter-diffusion barrier for Pd/ceramic/Ti-Al alloy composite membranes.

### Characterization of Pd/Ceramic/Ti-Al Alloy Composite Membranes

**Figure 4:**
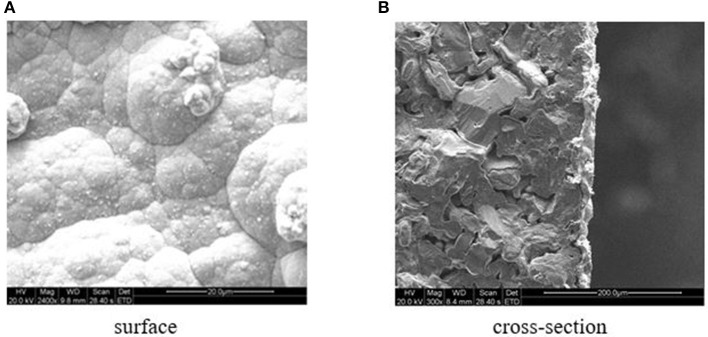
**(A)** Surface. **(B)** Cross-section. SEM photographs of the Pd/ceramic/Ti-Al alloy composite membrane.

The cross-sectional and surface SEM images of the Pd/ceramic/Ti-Al alloy composite membranes are shown in Figure 4. Figure 4A shows that the surface of the Pd membrane was not very smooth and homogeneous (mainly because of the high surface roughness of the ceramic/Ti-Al alloy support); however, no pinholes or cracks were observed. Although the images show smaller areas of the membranes, the gas leak test results further confirmed that the membranes had no defects at room temperature. Figure 4B shows that the thickness of the Pd membranes was uniform and was ~11–14 μm, which is consistent with the value determined by the gravimetric method (average thickness = ~ 13 μm).

### Gas Permeation of Pd/Ceramic/Ti-Al Alloy Composite Membranes

**Figure 5:**
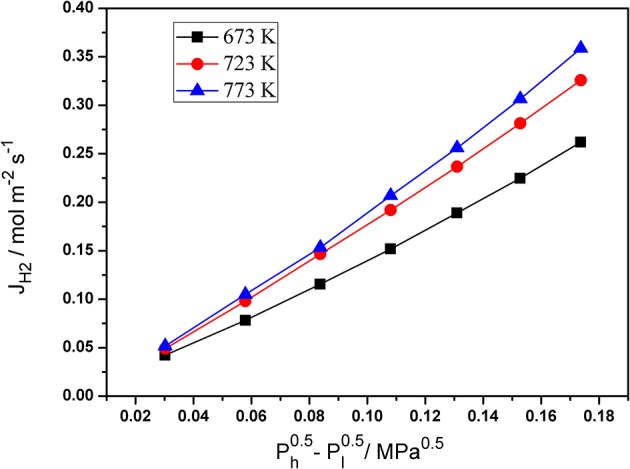
The dependence of hydrogen flux of Pd/ceramic/Ti-Al alloy composite membrane on $P_{h}^{0.5}$-$P_{l}^{0.5}$ at various temperatures.

Single gas permeation is an effective and simple method to measure the N_2_ and H_2_ permeation of palladium composite membranes. The pressure dependence of the hydrogen flux of the Pd composite membranes at various temperatures is shown in Figure 5. The H_2_ permeation flux increased with an increase in the pressure difference $\left(P_{h}^{0.5}-P_{l}^{0.5}\right)$. The temperature also had an obvious effect on the H_2_ permeation flux. These phenomena can be caused by the solution-diffusion mechanism.

**Figure 6:**
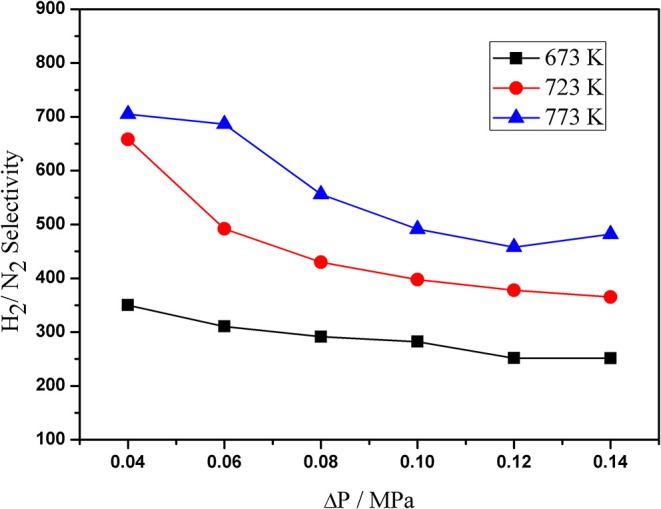
H_2_/N_2_ selectivity as a function of the transmembrane pressure at different temperature.

Figure 6 shows the H_2_/N_2_ selectivity of the Pd composite membranes at different pressure and temperature. The results show that the selectivity increased with an increase in the temperature from 673–773 K. The H_2_/ N_2_ selectivity was found to be within the range of 200–700 under the operating conditions. This can be attributed to the thin Pd layers and the high surface roughness of the support.

Table 3 shows a comparison of the membranes prepared in this study with those reported previously. The previously reported membranes had a thickness of ~20 μm and a hydrogen permeance coefficient of ~10^−3^-10^−4^ mol m^−2^ s^−1^ Pa^−0.5^. On the other hand, the hydrogen permeance coefficient of the membranes prepared in this study was of the order of 10^−3^ mol m^−2^ s^−1^ Pa^−0.5^. Hence, the selectivity of these membranes should be improved further.

**Table 3:** Comparison of the membrane prepared in this study and similar studies in the literatures.

**Membrane**	**Pore size of support (μm)**	**Thickness (μm)**	**Temperature (°C)**	***n***	**F mol m^**−2**^ s^**−1**^ Pa^**−0.5**^**	**Separation factor**	**References**
PSS/CeO_2_/Pd	0.2	13	500	0.5	1.27 × 10^−3^	∞	Tong et al., 2005
PSS/Oxide/Pd	0.2	21.3	450	0.5	1.43 × 10^−4^	∞	Rothenbergera et al., 2004
PSS/WO_3_/Pd	0.2	12	500	0.5	2 × 10^−3^	10,000	Zahedia et al., 2009
PSS/NaAZ80/Pd	0.2	19	450	0.5	1.1 × 10^−3^	608	Bosko et al., 2009
PSS/Al_2_O_3_/Pd	0.2	5	450	0.5	2.48 × 10^−3^	∞	Li et al., 2007
Ti-Al/TiO_2_/Pd	0.28	14	500	0.5	1.07 × 10^−3^	∞	Zhang et al., 2012
Ti-Al/ceramic/Pd	0.44	13	500	0.5	2.13 × 10^−3^	600	This work

Activation energy (E) is another critical parameter affecting the membrane permeability. The relationship between the hydrogen permeance of membranes and temperature can be shown by the Arrhenius equation as:

$$\begin{array}{l} Q\;=\;Q_{0}exp\left({-E}_{a}/RT\right) \end{array}$$

**Figure 7:**
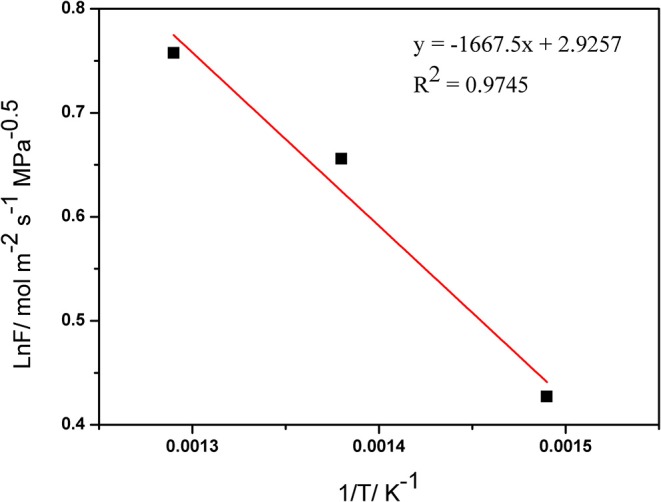
Arrhenius relation between the hydrogen permeance and temperature.

The Arrhenius relation between temperature and the hydrogen permeance is shown in Figure 7. The average activation energy for H_2_ permeation of the palladium composite membranes was calculated to be 13.86 kJ·mol^−1^ from 673–773 K, which is in agreement with the previously reported value. Table 4 lists the apparent activation energy of various membranes. These apparent activation energies were quite dispersed, due to various factors such as temperature, pressure, membrane structure, and other factors. Although the thicknesses of the Pd membranes were similar, different Pd membranes had different compositions, physical and chemical structures, and apparent activation energies.

**Table 4:** Comparisons of activation energy of different palladium membranes.

**Membrane**	**Thickness (μm)**	**Activation energy (kJ/mol)**	**Temperature (°C)**	**References**
Pd foil	50	11.4	350–600	Jung et al., 2000
Pd/PSS	20	16.38	350	Mardilovich et al., 1998
Pd/Al_2_O_3_	15	10	350	Dittmeyer et al., 2001
Pd/Al_2_O_3_	2.4–6	12.7–18.5	330–450	Keuler et al., 2002
Pd/TiO_2_	0.3–0.4	21.27	500	Wu et al., 2000
Pd/TiO_2_/Ti-Al	14	13.65	350–500	Zhang et al., 2012
Pd/ceramic/Ti-Al	13	13.86	400–500	This work

### Hydrogen Permeation Stability

**Figure 8:**
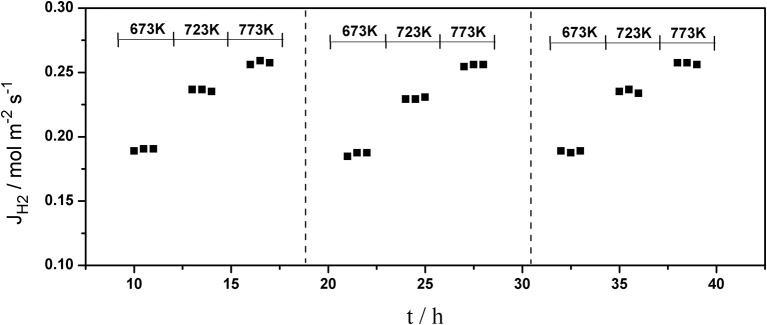
Variation of the hydrogen flux of Pd composite membrane during thermal cycling (ΔP = 0.1 MPa).

**Figure 9:**
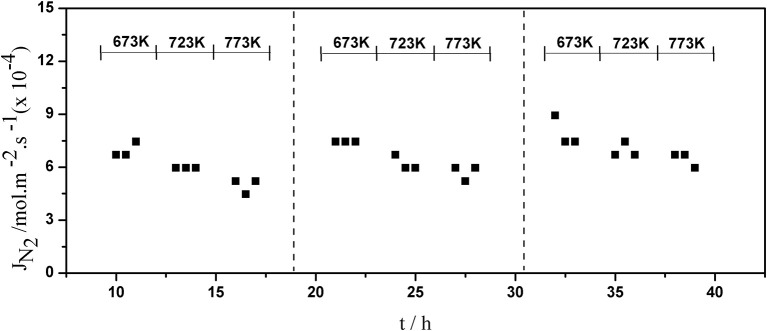
Variation of the nitrogen flux of Pd composite membrane during thermal cycling (ΔP = 0.1 MPa).

**Figure 10:**
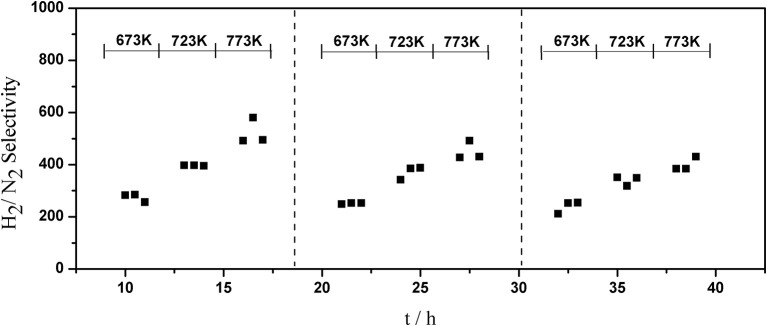
Variation of the H_2_/N_2_ selectivity of Pd composite membrane during thermal cycling (ΔP = 0.1 MPa).

The long-term stability of Pd composite membranes is crucial for practical applications. To investigate the effect of temperature variations on the H_2_ permeation of the composite membranes, temperature cycling was performed. Figures 8–10 show the hydrogen flux, nitrogen flux, and H_2_/N_2_ selectivity of the Pd/ceramic/Ti-Al composite membranes operating in three heat cycles of 673 K → 723 K → 773 K → 723 K → 673 K(at ΔP = 0.1 MPa). Under the same operating conditions, the H_2_ flux, the N_2_ flux, and H_2_/N_2_ selectivity of the Pd/ceramic/Ti-Al composite membranes showed no significant changes during the three heat cycles from 673–773 K, indicating that the Pd/ceramic/Ti-Al composite membranes possessed good thermal stability. This also shows that the ceramic layers were stable and could effectively avoid the inter-diffusion of metals.

## Conclusions

Ceramic membranes fabricated on porous Ti-Al alloy supports by an *in situ* oxidation method were used as the barrier layer between a palladium layer and a Ti-Al alloy disc to avoid intermetallic diffusion. The stabilities of the ceramic/Ti-Al alloy at high temperature were investigated in an H_2_ atmosphere, using gravimetric analysis, gas flux measurements, SEM, EDX, and XRD. The results showed that the thickness, pore size, and microscopy structure of the ceramic membrane were stable, even after heat treatment for 20 h at temperatures ranging from 773–973 K in an H_2_ atmosphere. This suggested that these ceramic layers are promising inter-diffusion barriers. Pd membranes were prepared on the ceramic/Ti-Al alloy substrates by using the electroless plating method. The average thickness and permeability of the membranes were ~13 μm and 2.13 × 10^−3^ mol m^−2^ s^−1^ Pa^−0.5^, respectively. The activation energy for H_2_ permeation was 13.86 kJ·mol^−1^ from 673–773 K. Heat cycling was performed to examine the stability of the composite membranes. The H_2_ flux, N_2_ flux, and H_2_/N_2_ selectivity of the membranes showed no observable changes after three heat cycles from 673–773 K, indicating that the Pd composite membranes had good thermal stability.

## Data Availability Statement

All datasets generated for this study are included in the article/supplementary material.

## Author Contributions

DZ and YF: conceptualization. JZ: data curation. PY: investigation. YC and JZ: methodology. DZ: writing and editing. All authors have read and agreed to the published version of the manuscript.

### Conflict of Interest

The authors declare that the research was conducted in the absence of any commercial or financial relationships that could be construed as a potential conflict of interest. The reviewer YJ declared a shared affiliation, though no other collaboration, with one of the authors YF to the handling editor.
